# Acknowledgement

**Published:** 2023

We are extremely grateful to the following members of the medical profession from Pakistan and Overseas for sparing their precious time to review the manuscript published in the Issue of July - August 2023.

**Table:** 

*Reviewer’s* *Name*	*(Manuscript* *Reviewed)*	*Reviewer’s* *Name*	*(Manuscript* *Reviewed)*	*Reviewer’s* *Name*	*(Manuscript Reviewed)*
Adeel Khalid, ***Pakistan***		Lu Wang, ***China***		Strazzi Sayhon, ***Brazil***	
Afzal Javed, ***UK***		Lubna Riaz, ***Pakistan***		Sultan Abdulwadood	
Ahmad Zaheer, ***Pakistan***		M Faheem Afzal, ***Pakistan***		Alshoabi, ***Saudi Arabia***	
Ahmed Badar, **UAE**		M Naeem Afzal, ***Pakistan***		Syed Fawad Mashhadi, ***Pakistan***	
Ali Akdemir, ***Turkey***		M. Bilal Mirza, ***Pakistan***		Syed Mamun Mahmud, ***UAE***	
Ali Hiader, ***USA***		Malik Abid Ali, ***Pakistan***		Syed Riazul Hasan, ***Pakistan***	
Ali Madeeh Hashmi, ***Pakistan***		Maria Sarwar, ***UK***		Tian-Yu Han, ***China***	
Alimova Sekina, ***Russia***		Masood Jawaid, ***Pakistan***		Umm-e-Kalsoom, ***Pakistan***	
Anita Haroon, ***Pakistan***		Meharunnisa Khaskheli, ***Pakistan***		Waris Qidwai, ***Pakistan***
Bushra Anwar, ***Pakistan***		Minwei Bao, ***China***		Xiao-fang Li, ***China***
Chen-yang Jiang, ***China***		Mohammad Sarwar Jamal, ***USA***		Xiao-jing Zhu, ***China***	
Chong-chao Yan, ***China***		Muhammad Ashraf		Xiaoxia Shen, ***China***	
Ebru KARAKOC, ***Turkey***		Ganatra, ***Pakistan***		Xudong Wang, ***China***	
Faisal Nazeer Hussain, ***Pakistan***		Muhammad Asif, ***Pakistan***		Yan Gao, ***China***	
Fang Ji, ***China***		Muhammad Khizar Niazi, ***Pakistan***		Zaheen Sibli, ***Pakistan***	
Farrukh Javeed, ***Pakistan***		Muhammad Perwaiz Iqbal, ***Pakistan***		Zahid Khan, ***Pakistan***	2
Feilong Wang, ***China***		Muhammad Saleem, ***Pakistan***		Zhi-gang Lang, ***China***	
Feroz Memon, ***Pakistan***		Muhammad Yasir Khan, ***Pakistan***		Zhi-hong Li, ***China***	
Gohar Wajid, ***Egypt***		Muhammad Zeeshan		Ziauddin A Shaikh, ***UK***	
Guo-rui Wang, ***China***		Sarawar, ***Pakistan***		Zohra Khanum, ***Pakistan***	
Hai-bo Li, ***China*** Haseeb Mehmood	*2*	Mukhtar Mehboob, ***Pakistan*** Muneeza Natiq, ***Pakistan***			
Qadri, ***Pakistan***		Musarrat Riaz, ***Pakistan***	8		
Humaira Zafar, ***Pakistan***	3	Mustafa Naeem, ***Pakistan***			
Ilkay Ceylan, ***Turkey***		Nazish Imran, ***Pakistan***			
Imran Ijaz Haider, ***Pakistan***	2	Noor-i-Kiran Haris, ***Pakistan***			
Irum Aamer, ***Pakistan***		Paul Sharpe, ***UK***			
Jiang Zheng, ***China***		Quan Wang, ***China***			
Jing Bi, ***China***		Rajia Liaqat, ***Pakistan***			
Jun Zang, ***China***		Ramesh Kumar, ***Pakistan***	2		
Kai Li, ***China***		Sahrai Saeed, ***Norway***	2		
Kai-long Liu, ***China***		Saima Askari, ***Pakistan***			
Khalid Waheed, ***Pakistan***		Samiullah Khan, ***Pakistan***			
Kashif Ahmed, ***USA***		Shankar Lal, ***Ireland***			
Li Chen, ***China***		Shaukat Ali Jawaid, ***Pakistan***			
Ling Zhang, ***China***		Sher Mohammad Khan, ***Pakistan***			

